# Importance of PTM of FLT3 in acute myeloid leukemia

**DOI:** 10.3724/abbs.2024112

**Published:** 2024-06-24

**Authors:** Jianwei Liu, Jianguo Gu

**Affiliations:** Division of Regulatory Glycobiology Institute of Molecular Biomembrane and Glycobiology Tohoku Medical and Pharmaceutical University 4-4-1 Komatsushima Aoba-ku Sendai Miyagi 981-8558 Japan

**Keywords:** FLT3, glycosylation, ubiquitination, cellular signaling, AML

## Abstract

FMS-like tyrosine kinase 3 (FLT3) is a receptor tyrosine kinase expressed in hematopoietic cells. Internal-tandem duplication domain (ITD) mutation and tyrosine kinase domain (TKD) mutation are the two most common mutations in acute myeloid leukemia (AML). Post-translational modifications (PTMs) of FLT3, such as glycosylation and ubiquitination, have been shown to impact various aspects of the protein in both wild-type (WT) and mutant forms of FLT3. In this review, we describe how the glycosylation status of FLT3 affects its subcellular localization, which significantly impacts the activation of downstream signaling, and the impact of specific ubiquitination on FLT3 function and stability, which may be associated with disease progression. Moreover, potential novel therapeutic strategies involving a combination of FLT3 tyrosine kinase inhibitors and drugs targeting glycosylation or ubiquitination are discussed.

## Introduction

Acute myeloid leukemia (AML) is a hematological malignancy originated from hematopoietic stem cells [1]. While relatively uncommon, comprising only 1% of all cancer diagnoses, AML is the predominant form of leukemia in the adult population [2]. The estimated 5-year overall survival rate is 30%, exhibiting significant variation across age groups, reaching 50% in younger patients and decreasing to below 10% in patients older than 60 years [3]. Several risk factors, including myelodysplastic syndrome (MDS), myeloproliferative disease, environmental exposures, and genetic predispositions, play a role in the pathogenesis of AML [2]. It is characterized by the clonal expansion of immature myeloid blast cells in the peripheral blood and bone marrow, leading to ineffective erythropoiesis and bone marrow failure [4]. With recent advancements in management guidelines, the overall cure rate has improved in younger patients. Although therapeutic regimens have advanced, the prognosis for the elderly population remains poor [5]. Mutations in genes involved in hematopoiesis are a significant feature of AML. Among them, FMS-like tyrosine kinase 3 (FLT3) is the most common mutation in AML [6]. The FLT3 protein belongs to the type III receptor tyrosine kinase (RTK) family, which is exclusively expressed in normal bone marrow stem/progenitor cells [7]. FLT3 is activated by binding of the FLT3 ligand (FL). Following activation, homodimers are assembled in the plasma membrane, resulting in receptor autophosphorylation. Activated FLT3 phosphorylates various effector molecules involved in the proliferation, differentiation, and apoptosis of hematopoietic cells. Approximately 30% of AML patients harbor constitutively activating mutations in the FLT3 gene, mostly internal tandem duplications (ITDs), which represent the insertion of nucleotide sequences of different lengths and at different sites [8]. The other predominant point mutation is the tyrosine kinase domain (TKD) mutation [9]. Papaemmanuil *et al*. [6] conducted a comprehensive study involving 1540 patients diagnosed with AML and reported the presence of FLT3 mutations in more than 500 patients. Christian *et al*. [10] performed a study involving 1485 patients diagnosed with AML and reported that the frequency of FLT3-ITD mutations was 312 of 1485 (21%). Bacher *et al*. [11] analyzed the mutational status and clinical significance of FLT3-TKD. They investigated 3082 patients with AML and observed FLT3-TKD mutations in 147 patients (4.8%). Furthermore, Ozeki *et al*. [12] reported that even in patients with wild-type (WT) FLT3, a discernible increase in overall survival was found in patients with high FLT3 expression (5 of 86 patients without FLT3-ITD mutations).

Among the various biological macromolecules, proteins display the most incredible diversity in function and structure. The gene-encoded primary polypeptide sequences of proteins primarily determine the structural and functional diversity of the cell proteome. However, this fundamental framework is significantly enhanced by alternative splicing of transcripts and a diverse range of post-translational modifications (PTMs). PTMs involve the covalent attachment of specific chemical groups to amino acid side chains through enzymatic or non-enzymatic processes [13]. PTMs have the potential to impact various facets of protein function, such as activity, subcellular localization, protein stability, intracellular signaling pathways, gene expression regulation, and cell-matrix interactions [14]. This review primarily focuses on PTMs of FLT3 in acute myeloid leukemia.

## Structure and Function of FLT3-WT and Its Mutants

**Figure 1:**
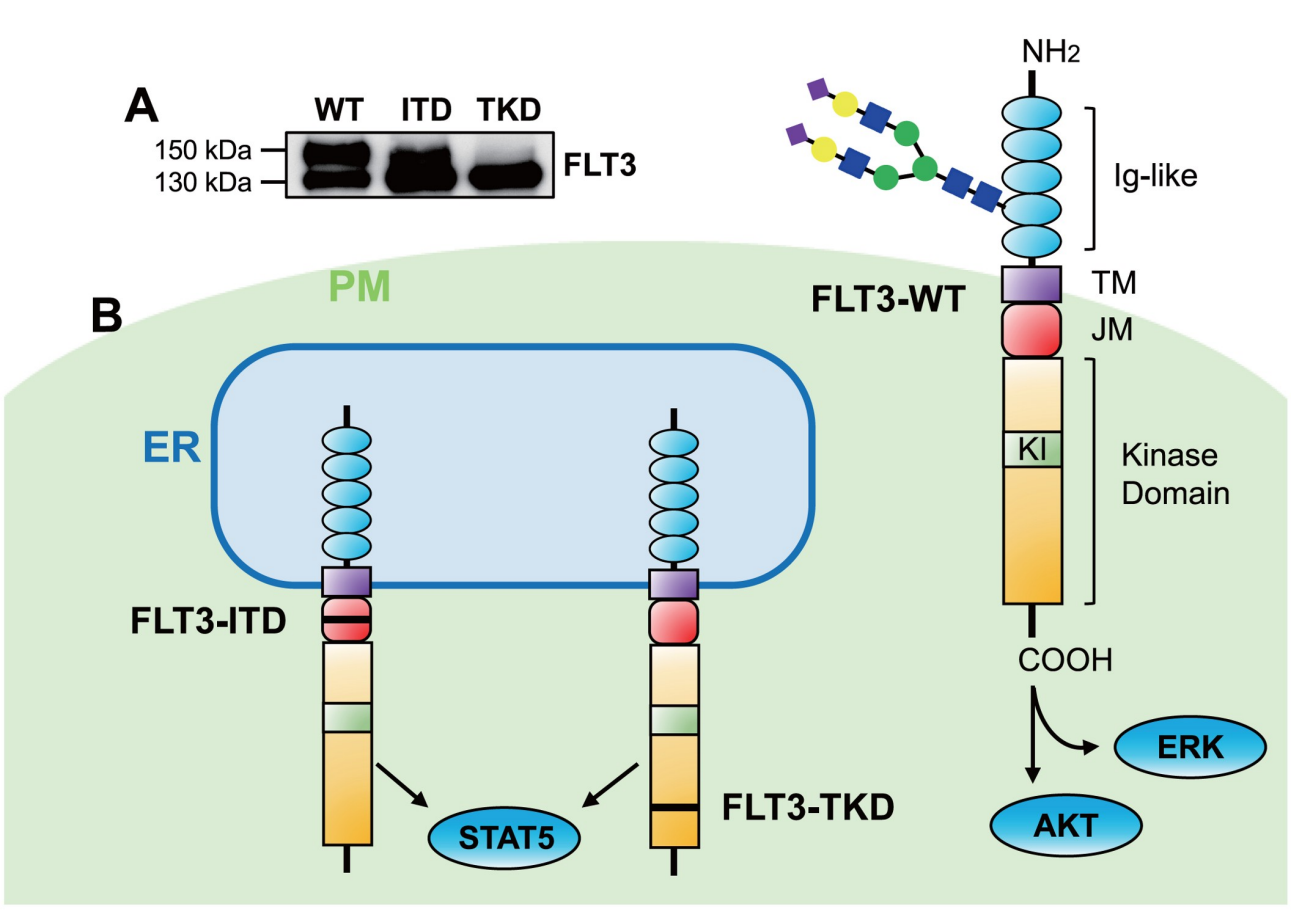
Figure 1 Structure, localization, and cellular signaling of FLT3 (A) Western blots showing different patterns of wild-type and mutant FLT3 due to glycosylation. (B) FLT3-WT is expressed on the cell surface to activate the MAPK and AKT signaling pathways, while FLT3-ITD and FLT3-TKD are located mainly in the ER, where they activate the STAT5 signaling pathway. FLT3 contains five distinct components: an extracellular immunoglobulin-like (Ig-like) domain containing N-glycans, a transmembrane domain (TM), a juxtamembrane domain (JM), and a kinase insert (KI). The black bar (‒) indicates an internal tandem duplication (ITD) in FLT3-ITD or a tyrosine kinase domain (TKD) mutation in the FLT3-TKD mutant.

There are two known forms of the FLT3 receptor according to western blot analysis. One is a mature form at approximately 150 kDa, which is thought to be complex glycosylated and expressed on the cell surface to activate mitogen-activated protein kinase (MAPK) signaling pathways. The other is an immature form at approximately 130 kDa, which represents an oligomannose form [15]. FLT3 has a protein structure similar to that of other type III receptor tyrosine kinases, featuring a kinase domain in the intracellular domain and immunoglobulin-like domains in the extracellular region. Specifically, the FLT3 receptor is composed of five distinct components ( Figure 1): an extracellular domain, a transmembrane region (TM), a juxtamembrane domain (JM), and a cytoplasmic tyrosine kinase domain (TKD), which are separated into two parts by a short region called the kinase insert (KI) [16]. The JM domain is composed of 3 topological parts: the JM-binding motif (JM-B), the switch motif (JM-S), and the linker/zipper peptide segment (JM-Z) [17]. JM-B makes contact with structural components implicated in the activation/inactivation cycle of the FLT3 cytoplasmic domain. JM-S is related to the activation and regulation of the FLT3 receptor enzyme. JM-Z can rotate around its attachment sites [ 17, 18].

FLT3-ITD mutation in the JM domain causes constitutive activation of tyrosine kinases, leading to autophosphorylation of the receptors and subsequent activation of downstream signaling pathways such as signal transducers and activators of transcription 5 (STAT5), ultimately facilitating the progression of leukemia [ 16, 19, 20]. According to a recent risk stratification analysis of patients with AML, those harboring FLT3-ITD mutations exhibited a decreased likelihood of attaining complete remission and were at increased risk of disease relapse [21]. According to the updated ELN classification (2022), AML patients with FLT3-ITD mutations are classified into the intermediate-risk group [22]. In addition to the common ITD mutation, FLT3-TKD is found in 5% to 7% of AML patients. The predominant mutation observed is the substitution of the 835 aspartic acid residue [ 23, 24]. Several recent studies have demonstrated that posttranslational modifications associated with FLT3 mutations and their cellular localization can result in alterations in downstream signaling pathways [25]. FLT3-ITD and TKD mutations have been shown to induce receptor phosphorylation in the absence of ligands, thereby facilitating AML progression through the activation of STAT5 signaling [ 16, 19, 26].

## FLT3 Glycosylation

Glycosylation, a commonly occurring posttranslational modification of proteins, has been associated with various physiological and pathological processes, including haematopoiesis and tumorigenesis [ 27‒ 29]. Numerous studies have demonstrated that glycosylation plays a vital role in protein folding, transport, location, dimer formation, and drug resistance [ 30‒ 34]. The N-glycosylation of FLT3 has garnered increasing attention.

### Glycosylation status of FLT3-WT and its mutants

FLT3 is initially synthesized as a 110 kDa protein lacking glycan modifications and subsequently undergoes preliminary glycosylation in the endoplasmic reticulum (ER) to form a 130 kDa immature protein containing oligomannose. This immature protein is further processed in the Golgi apparatus to produce a 150 kDa complex-glycosylated protein, which can ultimately be transported to the cell surface ( Figure 1) [15]. The 150 kDa form is more prominent in the FLT3-WT cells than in the ITD and TKD mutant cells. In FLT3-ITD cells, the majority of FLT3 was found to be in the 130 kDa family, with a minor fraction in the mature family, whereas FLT3-TKD cells predominantly exhibit the 130 kDa family [ 15, 35]. The precise mechanism underlying the lower glycosylation levels in FLT3 mutants remains to be defined. Natarajan *et al*. [36] indicated that the interaction with the oncogenic serine/threonine kinase Pim-1 and subsequent serine phosphorylation by Pim-1 might contribute to the stabilization of the 130 kDa FLT3-ITD, preventing its further modification by complex types of glycans.

**Figure 2:**
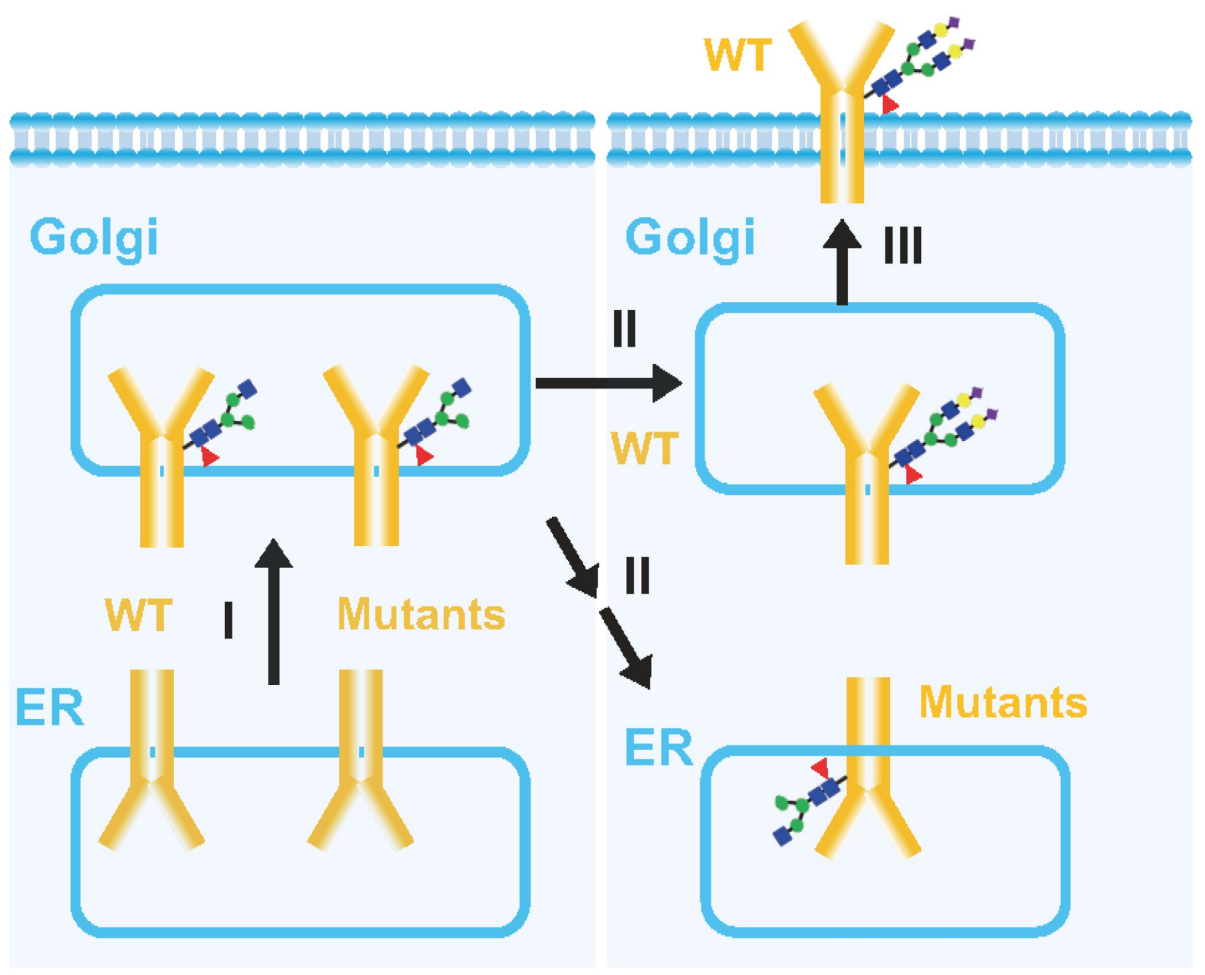
Figure 2 Proposed transport modes of FLT3-WT and the mutants First, FLT3-WT and its mutants are processed in the ER. After primary After primary modification with oligomannose, they are transported to the Golgi apparatus for complex glycosylation (I). FLT3-WT is further processed in the Golgi for complete complex N-glycosylation (II) and is finally transported and expressed on the cell surface (III). On the other hand, the FLT3 mutants undergo light N-glycosylation, such as with hybrid N-glycan status, and are reversely transported back to the ER (II) through undefined mechanisms.

It has been reported that *Galanthus nivalis* lectin (GNA), a selective lectin with a mannose-rich structure [37], interacts specifically with the 130 kDa species of both FLT3-WT and FLT3-ITD [15], which suggests the presence of oligomannose types on FLT3-ITD. However, it was also reported that the 130 kDa form of FLT3-ITD or TKD, but not WT, contains core fucosylation confirmed by *Aleuria aurantia* lectin (AAL) [38], a lectin that preferentially recognizes core fucosylated N-glycans catalyzed by α-1,6-fucosyltransferase (FUT8) [ 39, 40]. Therefore, the glycosylation status of these cells differed between the FLT3-WT and FLT3-mutant groups. Although the mutants have been reported to mainly localize to the ER, they might be transported to the Golgi apparatus once and then reversely transported back to the ER through unknown mechanisms ( Figure 2).

The expression of either WT or mutant FLT3 in Ba/F3 cells, which are interleukin-3 (IL-3)-dependent hematopoietic progenitor cells, significantly induced core fucosylation [38]. Interestingly, *FUT8* deficiency ( *FUT8*-KO) induces cell growth in FLT3-WT Ba/F3 cells even without IL-3. Additionally, *FUT8*-KO significantly increases the phosphorylation of STAT5 and the activation of AKT and ERK without affecting the localization of FLT3-WT. A possible mechanism is that the deficiency of core fucosylation on FLT3 facilitates the dimerization of FLT3, leading to the aberrant activation of FLT3-WT-mediated signaling [38]. These findings indicate that core fucosylation is a critical factor in FLT3 signaling and provides a valuable avenue for treating AML. In contrast, ablation of FUT8 partially inhibits the proliferation of ITD and TKD cells, suggesting that glycan-mediated signaling is quite different between the WT and mutant strains.

### Abnormal localization and cellular signaling of FLT3 caused by protein-protein interactions

Accumulating evidence suggests that the glycosylation level of FLT3 and dynamic changes in its subcellular localization significantly impact the activation of downstream signaling pathways. FLT3-WT is mainly localized on the cell surface and interacts with FL to be activated [41]. The combination of FLT3-WT with FL has been shown to activate several downstream signaling pathways, such as the PI3K/AKT and MAPK signaling pathways [ 42, 43]. In contrast, FLT3-ITD receptor proteins predominantly accumulate in the perinuclear region [ 44, 45]. Kouhei Yamawaki *et al*. [46] reported that the mutant FLT3-ITD protein is primarily localized in the ER and Golgi apparatus, with a minority fraction present at the plasma membrane. The differential location of FLT3-ITD mutations is related to differences in signal quality. FLT3-ITD localized at the cell surface strongly activates the MAPK and PI3K/AKT pathways. In contrast, FLT3-ITD localized at the ER aberrantly activates STAT5 but cannot upregulate the MAPK and PI3K/AKT pathways [ 47, 48]. Furthermore, plasma membrane-resident FLT3-ITD was also observed to activate K-Ras in AML cells [49]. Compared with FLT3-ITD, ER-anchored FLT3-TKD also weakly activates STAT5 ( Figure 1) [50]. Recent research indicated that the tyrosine kinase inhibitors quizartinib (AC220) and midostaurin (PKC412), both known to effectively inhibit FLT3 [ 51‒ 53], increase the plasma membrane distribution of FLT3-ITD [46]. Consistently, Reiter *et al*. [45] observed that treatment with AC220 resulted in a shift in the intracellular localization of FLT3-ITD and FLT3-TKD to a plasma membrane localization resembling that of FLT3-WT. Inhibition of the FLT3-ITD kinase by small molecules, inactivating point mutations, or co-expression with protein-tyrosine phosphatases enhances complex glycosylation and surface localization of the FLT3-ITD [15]. These findings serve as the foundation for the potential synergistic effect of combining a tyrosine kinase inhibitor with FLT3-directed immunotherapy in treating FLT3-ITD-positive AML patients.

The mechanisms underlying the changes in the subcellular localization of FLT3 resulted from activating mutations are receiving increasing attention. The first explanation is that the nucleophosmin-1 (NPM1) mutation (NPM1c) causes the mislocalization of FLT3-TKD and changes its signal transduction ability [54]. NPM1 mutation is another common gene aberration in AML patients [55]. NPM1c induces a shift in the subcellular localization of FLT3-TKD from the plasma membrane to the ER, facilitating the activation of STAT5 induced by FLT3-TKD. Moreover, aberrant STAT5 activation is observed in primary murine cells and AML patients harboring concurrent FLT3-TKD and NPM1c mutations.

Marcotegui *et al*. [56] recently revealed that the SET protein is involved in FLT3 trafficking. As a scaffold protein, SET co-localizes with FLT3 and facilitates the transport of nascent FLT3 to the cell membrane in FLT3-WT cells. SET nuclear retention decreased the amount of FLT3-WT on the membrane, underscoring the critical role of SET in FLT3 membrane trafficking. In contrast, the FLT3-ITD mutation impairs SET/FLT3 binding, contributing to its retention in the ER. When FLT3-WT cells were treated with tunicamycin, an inhibitor of protein glycosylation, the amount of FLT3 in the membrane significantly decreased, while the colocalization of SET/FLT3 remained unchanged. This finding implies that the SET/FLT3 interaction occurs before N-glycosylation. Furthermore, they suggested that the FLT3 inhibitor PKC412 facilitates the translocation of FLT3-ITD to the cytoplasmic membrane by enhancing the binding between FLT3 and SET.

Rai *et al*. [57] reported that clathrin assembly lymphoid myeloid leukemia protein (CALM) disrupts the intracellular localization of FLT3-ITD, consequently inhibiting its cellular signaling. *CALM* knockdown or chlorpromazine (which can reduce CALM protein expression) treatment reduced the co-localization of FLT3 ITD with the ER and inhibited the phosphorylation of STAT5. Additionally, studies have shown that chaperone proteins such as calreticulin, calnexin, and HSP90beta1 play direct roles in the retention of FLT3-ITD in the ER [34]. These results may provide insights into the mechanisms underlying the intracellular retention of FLT3 mutants.

### Targeting drug therapy for FLT3 glycosylation

FLT3 inhibitors are widely utilized for the treatment of AML, significantly improve survival and have demonstrated notable enhancements in the survival rates and prognoses of individuals with AML [58]. However, the efficacy of FLT3-targeted tyrosine kinase inhibitors has been compromised by the development of adaptive and acquired resistance via various distinct mechanisms [59]. These limitations warrant the development of novel, targeted agents. The altered localization of FLT3 mutants caused by different glycosylation statuses affects their downstream signaling pathways, thus prompting increased interest in the investigation of glycosylation inhibitors as a potential therapeutic avenue for AML.

2-Deoxy-D-glucose (2-DG) is recognized as a sugar analogue that inhibits N-linked glycosylation. Due to its potent inhibition of the glycolytic pathway, the anticancer properties of 2-DG have been extensively investigated in solid tumors [ 60, 61]. According to previous reports [62], 2-DG inhibits cell viability and induces apoptotic cell death in FTL3-ITD-positive cells. Through N-linked glycosylation inhibition, 2-DG affects the cell surface expression and cellular signaling of FTL3-ITD. Moreover, the effect of 2-DG was also demonstrated in quizartinib-resistant FLT3-TKD cells. Since leukemia cells harboring FLT3-ITD mutations are more sensitive to 2-DG treatment than wild-type cells, 2-DG may serve as a promising therapeutic target for AML patients with mutated FLT3.

Statins were developed to lower cholesterol and triglyceride levels in the body. This is achieved through the inhibition of 3‑hydroxy-3-methylglutaryl coenzyme A (HMG CoA) reductase, a crucial enzyme that regulates the mevalonate pathway [63]. The mevalonate pathway is also involved in the biosynthesis of dolichol, which is related to the co-translational transfer of oligosaccharides to nascent polypeptides through N-linked glycosylation [64]. Previous research has shown that fluvastatin impairs the glycosylation of FLT3 and its transport to the cell surface of FLT3-ITD cells [59]. Fluvastatin has the potential to induce cytotoxic effects in mutant FLT3 leukemia cell lines through the disruption of signal transduction pathways, thus reducing tumor burden *in vivo*. Significantly, fluvastatin treatment effectively overcomes several resistance mechanisms, making it a potential therapeutic option for treating patients with FLT3-mutant AML.

Tunicamycin is a bacterial antibiotic that effectively inhibits the process of active sugar transfer to dolichol phosphate, which is a crucial step in the formation of N-glycosylated proteins in the ER [65]. As an inhibitor of N-linked oligosaccharide synthesis, tunicamycin has been demonstrated to induce cytotoxic effects and increase the sensitivity of various cancer cells to therapy [ 66, 67]. Tsitsipatis *et al*. [68] reported that inhibiting the maturation of the FLT3-ITD glycoprotein using low concentrations of tunicamycin had antiproliferative and proapoptotic effects on FLT3-ITD-expressing human and murine cell lines [68]. Low doses of tunicamycin significantly attenuate AKT and ERK activation in FLT3-ITD-positive cells. Tunicamycin demonstrated significant efficacy in reducing the viability of leukemia cell lines expressing FLT3-ITD and displayed synergistic effects with FLT3-ITD kinase inhibitors [68]. These findings suggest that a range of glycosylation inhibitors can decrease the glycosylation of FLT3 mutants, disrupting their plasma membrane expression and cellular signaling, providing novel insight for improving the therapeutic effect of AML treatment.

## FLT3 Ubiquitination

Another essential post-translational modification of FLT3 is ubiquitination. Ubiquitination is a process in which ubiquitin forms a bond with a lysine residue at the target site of a protein, playing a crucial role in regulating almost all cellular metabolism [69]. This process is dynamic and reversible and is regulated by ubiquitin ligases and deubiquitin enzymes (DUBs) [70]. Ubiquitin is covalently conjugated to substrates through a complex three-step enzymatic process involving E1 ubiquitin-activating enzymes, E2 ubiquitin-conjugating enzymes, and various E3 ubiquitin ligases [ 71, 72]. Deubiquitination is the process of hydrolysing ubiquitin from a substrate or polyubiquitin chain and is regulated by a deubiquitinase [73]. Based on the diverse ubiquitination sites, many linkages exist in ubiquitin chains. Among them, K48- and K63-linked polyubiquitin chains are the most extensively researched and prevalent forms in cells [74]. According to the traditional understanding of K48-linked polyubiquitination, the proteasome can interact with a chain consisting of at least four ubiquitin moieties to target polyubiquitinated substrates for degradation [75]. The K63 chain is involved in DNA damage repair, receptor trafficking, kinase signaling pathways, and ribosomal biogenesis [76]. The ubiquitination of FLT3 is triggered by the activation of FLT3 through ligand binding and is mediated by multiple ubiquitin ligases, ultimately regulating the internalization and degradation of the receptor [ 77, 78]. FLT3 was found to be poly-ubiquitinated through both K48 and K63 linkages [16].

### K48-linked ubiquitination of FLT3

As mentioned above, K48-linked chains are the most prevalent chain type and target proteins for proteasomal degradation. Cbl facilitates K48-linked polyubiquitination of autophosphorylated FLT3-ITD, and Cbl-mediated K48-linked ubiquitination is recognized by the proteasome, resulting in the degradation of FLT3 in proteasomes [ 79‒ 81]. The Cbl proteins are a highly conserved family of RING finger E3 ubiquitin ligases that regulate signaling via receptor and nonreceptor tyrosine kinases [82]. Members of the Cbl family are recognized as significant receptor tyrosine kinase ubiquitination regulators. Overexpression of loss-of-function Cbl mutants results in increased activation of FLT3 downstream signaling pathways, such as AKT and STAT5, further demonstrating its role in the negative regulation of FLT3 signaling [83].

Weisberg *et al*. [84] identified USP10, a K48-linked ubiquitin deubiquitinase, as an essential DUB required to stabilize FLT3. Pharmacological inhibition of USP10 results in the degradation of FLT3 and demonstrates efficacy in FLT3-ITD-positive AML models, including cell lines, primary patients, and mouse models.

### K63-linked ubiquitination of FLT3

K63-linked chains are not involved in proteasomal degradation; however, they are crucial for the regulation of lysosomal functions and the internalization of membrane receptors [ 85, 86]. Both lysosomal and proteasomal degradation of FLT3 have been documented [ 79, 87]. K63-linked polyubiquitination of FLT3 has been reported to occur via neural precursor cell-expressed developmentally downregulated protein 4 (NEDD4) [79]. The deubiquitinase USP9X physically associates with FLT3-ITD, leading to the inhibition of its K63-linked polyubiquitination. The inhibition of USP9X by its inhibitor or USP9X-shRNA enhances K63-linked polyubiquitination of FLT3-ITD and induces apoptosis in FLT3-ITD-positive AML cells [88]. Downregulating USP9X can also reduce the expression levels of FLT3-ITD and its associated downstream signaling pathways, suggesting that USP9X represents a promising target for treating FLT3-ITD-positive AML patients.

**Figure 3:**
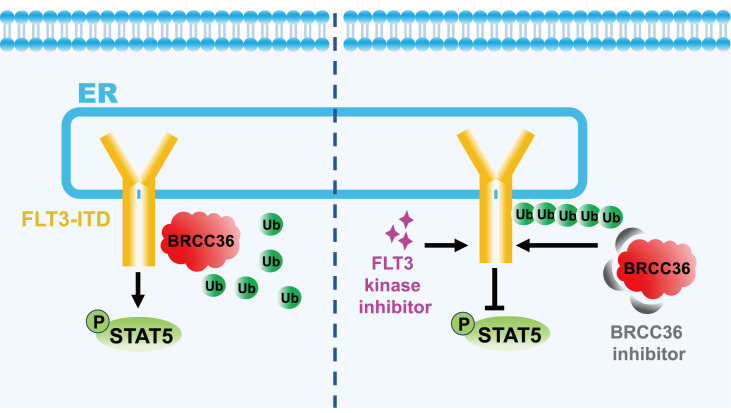
Figure 3 Synergistic effects of BRCC36 inhibitors and FLT3 kinase inhibitors on FLT3-ITD-mediated signaling BRCC36 hydrolyzes the K63-linked polyubiquitin chains of FLT3-ITD and promotes its protein stability. The BRCC36 inhibitor increases the number of K63-Ub chains on FLT3-ITD and induces its degradation. BRCC36 inhibitors and FLT3 kinase inhibitors have synergistic effects on suppressing FLT3-ITD activity.

Our recent study also demonstrated that K63-linked polyubiquitination plays a regulatory role in FLT3-ITD [35]. Using proximity labelling technology with TurboID, we found that BRCA1/BRCA2-containing complex subunit 36 (BRCC36), a specific K63-linked polyubiquitin deubiquitinase, was exclusively associated with ITD. TurboID is a novel proximity-dependent labelling technique that utilizes biotin and ATP to generate biotin-5'-AMP. This reactive intermediate can rapidly label lysine residues of proximal proteins. Biotinylated proteins are then digested, enriched, and identified. TurboID has higher activity, enabling higher temporal resolution and broader application *in vivo* [89]. Our results indicated the presence of K63-linked polyubiquitin on FLT3-ITD and showed that BRCC36 promotes its protein stability and activation by hydrolyzing K63-linked polyubiquitin chains. Moreover, it is worth noting that the BRCC36 inhibitor had synergistic effects with the FLT3 tyrosine kinase inhibitor for treating AML ( Figure 3). In general, attenuation of FLT3 stability by pharmacological modulation of ubiquitination has significant clinical advantages for patients with oncogenic FLT3-positive AML.

## Conclusion and Future Directions

Increasing research on FLT3 has significantly expanded our understanding of its biological function. FLT3-ITD mutation is a significant genetic alteration in AML and is a critical prognostic indicator for adverse outcomes in this hematologic malignancy. A distinct glycosylation status leads to the aberrant localization of mutant FLT3 in cells, triggering the aberrant signaling pathway. FLT3-WT is thought to be fully N-glycosylated and is then expressed on the cell surface to activate the AKT and MAPK signaling pathways. On the other hand, FLT3-ITD displays reduced glycosylation and mainly localizes to the ER, leading to efficient activation of STAT5. As described above, many factors contribute to the aberrant PTM and localization of FLT3 mutants.

To date, most of our current understanding of the downstream signaling pathway of FLT3 has been derived from studies utilizing transfected cell lines or cells from individuals with AML. Transgenic mice lacking specific signal transduction molecules or mice expressing FLT3 mutants have not yet been extensively used to study FLT3 function. Therefore, related work needs to be performed.

Given the significant role of FLT3 in AML, further elucidation of the underlying mechanisms is needed. Improving our understanding of signaling in individual AML patients is necessary. The different responses of patients to current selective kinase inhibitors may be attributed to variations in the microenvironment and epigenetic factors among the individual patients. In addition, considering the propensity of leukemia to develop resistance to FLT3 inhibitors, we need to gain a deeper understanding of the mechanism by which FLT3 signals in AML cells. This knowledge enables the identification of novel target molecules for drug development, potentially leading to the development of novel therapies.
